# Association Between Visual Impairment and Decline in Cognitive Function in a Multiethnic Asian Population

**DOI:** 10.1001/jamanetworkopen.2020.3560

**Published:** 2020-04-23

**Authors:** Zhi Wei Lim, Miao-Li Chee, Zhi Da Soh, Ning Cheung, Wei Dai, Thakur Sahil, Yijin Tao, Shivani Majithia, Charumathi Sabanayagam, Christopher Li-Hsian Chen, Tien Yin Wong, Ching-Yu Cheng, Yih-Chung Tham

**Affiliations:** 1 Singapore Eye Research Institute, Singapore National Eye Centre, Singapore; 2 Faculty of Medicine, University of New South Wales, Sydney, New South Wales, Australia; 3 Duke-NUS Medical School, Singapore; 4 Memory Aging and Cognition Centre, National University Health System, Singapore; 5 Department of Pharmacology, Yong Loo Lin School of Medicine, National University of Singapore and National University Health System, Singapore; 6 Department of Ophthalmology, Yong Loo Lin School of Medicine, National University of Singapore and National University Health System, Singapore

## Abstract

**Importance:**

With the rapidly aging population, the burden of visual impairment (VI) and cognitive decline is expected to increase. Previous cross-sectional studies suggest an association between these 2 health outcomes. However, few longitudinal reports have examined this association, and to our knowledge, no studies have been performed in Asian populations. Further investigation on this association may help to better identify individuals at risk of cognitive decline.

**Objective:**

To examine the longitudinal association between VI and decline in cognitive function in a multiethnic Asian population.

**Design, Setting, and Participants:**

In this longitudinal, population-based, prospective cohort study, Chinese, Indian, and Malay adults 60 years or older at baseline were recruited from the Singapore Epidemiology of Eye Diseases (SEED) study. At baseline, participants from the SEED study were recruited under 3 studies: the Singapore Malay Eye Study (SiMES; 2004-2006), the Singapore Indian Eye Study (SINDI; 2007-2009), and the Singapore Chinese Eye Study (SCES; 2009-2011). Eligible participants were reexamined after 6 years (2011-2013 for SiMES, 2013-2015 for SINDI, and 2015-2017 for SCES). Data analysis was performed from November 1 to 24, 2019.

**Exposures:**

Visual impariment was defined as presenting visual acuity worse than 20/40 based on the better-seeing eye.

**Main Outcomes and Measures:**

Cognitive function was assessed using a locally validated Abbreviated Mental Test (AMT). The association between baseline VI and change in AMT score was determined using the multivariable linear regression model adjusting for baseline age; sex; race/ethnicity; presence of diabetes, hyperlipidemia, hypertension, and chronic kidney disease; history of cardiovascular disease; smoking status; alcohol intake; body mass index; educational status; and AMT score.

**Results:**

A total of 2478 individuals (1256 [50.7%] male; 1073 Chinese, 768 Indian, and 637 Malay adults) with mean (SD) age of 67.6 (5.6) years were evaluated, of whom 489 (19.7%) had reduction in AMT scores over 6 years. Baseline VI was associated with a decrease in AMT score over 6 years (β = −0.27; 95% CI, −0.37 to −0.17; *P* < .001). When change in vision over 6 years was evaluated, unchanged or deteriorated VI was associated with a decrease in AMT score over 6 years (β = −0.29; 95% CI, −0.40 to −0.18; *P* < .001). Among individuals with baseline VI and a substantial decrease in AMT score of 3 units or more over 6 years, the leading causes of VI were undercorrected refractive error (14 [45.2%]) and cataract (11 [35.5%]).

**Conclusions and Relevance:**

In this study, poor vision was independently associated with a decline in cognitive function. Causes of visual loss in these cases were mostly preventable, further suggesting that preserving good vision may be an important interventional strategy for mitigating cognitive decline.

## Key Points

**Question:**

Was there an association between visual impairment and decline in cognitive function over a 6-year period in a multiethnic Asian population?

**Findings:**

In this population-based cohort study of 2478 elderly Asian people, visual impairment at baseline and deterioration of vision over time were associated with decline in cognitive function over 6 years. The main causes of visual impairment among individuals with substantial cognitive decline were undercorrected refractive error and cataract, both of which are usually preventable or treatable.

**Meaning:**

Early intervention of visual impairment in elderly individuals may potentially mitigate decline in cognitive function.

## Teaser

This cohort study examines the association between visual impairment and decline in cognitive function in a multiethnic Asian population in Singapore over a 6-year period.

## Introduction

Visual impairment (VI), defined as low vision or blindness, affects approximately 441.1 million individuals, disproportionately affecting individuals 50 years and older.^1^ With the rapidly aging populations,^2^ the prevalence of VI is expected to increase 3-fold by 2050.^1^ VI imposes a substantial health burden in terms of quality-adjusted life-years or years lived with disability that is comparable to major chronic diseases, such as diabetes, dyslipidemia, cardiovascular diseases (CVD), and obesity.^3,4^ Likewise, deficits in cognitive function are also a major cause of morbidity.^4^ Previous studies^5,6,7^ have found a close association between VI and decline in cognitive function.

It has been postulated that visually impaired individuals have more difficulties in performing daily activities, such as reading and seeing faces.^8^ Reduced engagement in these cognitively stimulating activities may lead to brain reserve deficits, resulting in decline in cognitive function.^9,10,11,12,13^ In this regard, several cross-sectional studies^12,14,15,16,17^ found a significant association between VI and decline in cognitive function. However, longitudinal reports^5,6,7,18^ in this area are currently scarce and nonconclusive. Furthermore, to our knowledge, no longitudinal studies have investigated the association between VI and cognitive decline in Asian populations.

The objective of this study was to determine the longitudinal association between VI and cognitive decline over time in a multiethnic, elderly Asian population. Findings from this study are intended to help better identify elderly Asian individuals who may be at higher risk of cognitive decline.

## Methods

### Study Population

We conducted a prospective, population-based cohort study using individuals recruited from the Singapore Epidemiology of Eye Diseases (SEED) study, which was composed of 3 major Asian ethnic groups in Singapore. At baseline, participants from SEED were recruited under 3 studies: the Singapore Malay Eye Study (SiMES; 2004-2006), the Singapore Indian Eye Study (SINDI; 2007-2009), and the Singapore Chinese Eye Study (SCES; 2009-2011). All study procedures were performed in accordance with the principles of the Declaration of Helsinki,^19^ and ethical approval was obtained from the Singapore Eye Research Institute Institutional Review Board. Written informed consent was obtained from all study participants. Data were deidentified before the analysis was performed. This study followed the Strengthening the Reporting of Observational Studies in Epidemiology (STROBE) reporting guideline.^20^

In brief, an age-stratified random sampling was used to select Malay, Indian, and Chinese adults aged 40 to 80 years. A total of 4168 Malay, 4497 Indian, and 4606 Chinese adults were randomly selected, of whom a total of 10 033 individuals (3280 Malay, 3400 Indian, and 3353 Chinese adults) participated in the study, with an overall response rate of 75.6%.

Follow-up examination of the SEED study was conducted 6 years later (2011-2013 for SiMES, 2013-2015 for SINDI, and 2015-2017 for SCES). The methods of the initial and follow-up SEED studies have been described in detail previously.^21,22,23,24^

In the baseline examination, cognitive assessment by the Abbreviated Mental Test (AMT) was performed only among participants 60 years or older (n = 4407). Participants with missing baseline AMT data (n = 97) or missing baseline visual acuity (VA) data (n = 1) were excluded. Of the remaining 4309 individuals from baseline, 1134 were ineligible for follow-up in the SEED study because of death (n = 652), migration or relocation (n = 113), and severe illness or severe mobility impairment (n = 369). Of the 3175 participants eligible for follow-up, 688 did not attend the follow-up examination. Consequently, 2487 individuals were reexamined on follow-up. Of these individuals, 9 did not complete the AMT, thereby leaving 2478 participants included in the final analysis (response rate, 78.0% [2478 of 3175]) (Figure).

**Figure.**
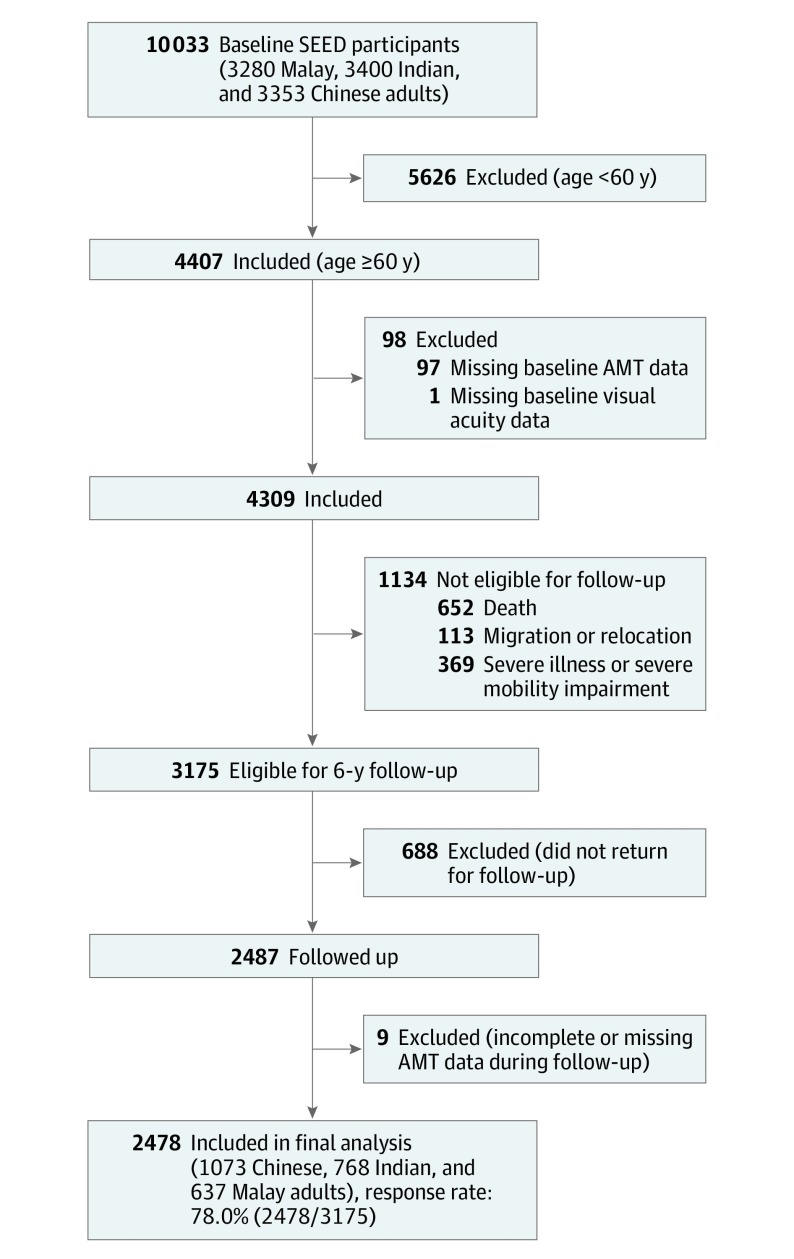
Flowchart for Inclusion and Exclusion of Study Participants AMT indicates Abbreviated Mental Test; SEED, Singapore Epidemiology of Eye Diseases.

### Vision Assessment

Presenting distance VA data were collected for both eyes. VA was measured at 4 m using the logMAR number chart (Lighthouse International). If the largest print of numbers could not be read at 4 m, the individuals were subsequently positioned at 3, 2, and 1 m. If no numbers were read correctly from the chart, VA was measured and recorded as counting fingers, hand movement, light perception, or no light perception accordingly. For presenting VA, participants were instructed to wear their own prescriptive correction, if any.

In this study, VI was based on the US definition.^25^ Any VI was defined as presenting VA worse than 20/40. Low vision was defined as presenting VA worse than 20/40 but better than 20/200, whereas blindness was defined as presenting VA worse than or equivalent to 20/200.

### Assessment of Cognitive Function

We assessed cognitive performance using the AMT (eFigure in the Supplement), a 10-question abbreviated questionnaire derived from the 26-question Roth-Hopkins test.^26,27^ The version of the AMT questionnaire used was slightly modified for the Singapore context^28^ and had been validated in several population-based studies.^16,29,30,31,32,33,34^ The AMT was administered by trained study investigators to participants 60 years or older in a language of their preference. All questions were of equal weight (1 mark each), culminating in a total possible score of 10. Change in AMT score over time was calculated as the AMT score at 6-year follow-up minus the AMT score at the baseline visit. A negative difference denotes a decrease in AMT score.

### Other Measurements and Systemic Assessments

For all participants at the baseline visit, we used interviewer-administered questionnaires to ascertain information about their demographic characteristics, lifestyle risk factors (ie, current smoking status and alcohol intake), and medical history. Educational level was categorized as no formal education; primary education; O levels or equivalent; A levels, polytechnic, diploma, or technical school equivalent; or university education. Diabetes was defined as a random glucose level of 200 mg/dL or higher (to convert to millimoles per liter, multiply by 0.0555), glycated hemoglobin level of 6.5% of total hemoglobin or greater (to convert to proportion of hemoglobin, multiply by 0.01), current use of diabetes medication, or self-reported history of diabetes. Hyperlipidemia was defined as a total cholesterol level of 239 mg/dL or higher (to convert to millimoles per liter, multiply by 0.0259) or current use of lipid-lowering medication. Hypertension was defined as systolic blood pressure of 140 mm Hg or higher, diastolic blood pressure of 90 mm Hg or higher, current use of blood pressure medication, or self-reported history of hypertension. Chronic kidney disease (CKD) was defined as estimated glomerular filtration rate less than 60 mL/min/1.73 m^2^ based on the Chronic Kidney Disease Epidemiology Collaboration equation.^35^ History of CVD was defined based on self-reported history of angina, stroke, or myocardial infarction. Body mass index (BMI) was calculated as weight in kilograms divided by height in meters squared and categorized as underweight (<18.5), healthy weight (≥18.5 to <25), overweight (≥25 to <30), or obese (≥30).

### Statistical Analysis

All statistical analyses were performed using Stata statistical software, version 13 (StataCorp LLC). Statistical significance was determined as *P* < .05 based on a 2-sided evaluation. Age, BMI, and AMT scores at baseline were analyzed as continuous variables, whereas sex; race/ethnicity; presence of diabetes, hyperlipidemia, hypertension, and CKD; history of CVD; alcohol intake; current smoking status; educational status; and VI were analyzed as categorical variables. We performed an independent, 2-tailed, unpaired *t* test for continuous data and Pearson χ^2^ test for categorical data to compare between individuals included and excluded in our final analysis and between individuals with and without presenting VI at baseline (based on better eye). In addition, we also compared baseline VI status between individuals with and without a decrease in AMT score over time.

A multivariable linear regression model was used to ascertain the associations between baseline presenting VA and VI with change in AMT score over 6 years. Model 1 was adjusted for baseline age, sex, and race/ethnicity, whereas model 2 was further adjusted for baseline presence of diabetes, hyperlipidemia, hypertension, and CKD; history of CVD; current smoking status; alcohol intake; BMI; educational status; and AMT score. Multivariable linear regression was performed separately for different baseline VI statuses: (1) VI based on better eye and (2) VI based on worse eye. For finer analysis of severity, VI was further categorized into low vision and blindness. Association between change in vision status (over 6 years) and change in AMT score was also evaluated. Sensitivity analyses excluding items 8 and 10 from the aggregate score of AMT were conducted for the associations between baseline vision and change in vision status with change in AMT score. We further evaluated the main causes of VI among individuals with baseline VI and a substantial decrease in AMT score of 3 units or more over the 6-year follow-up period. Data analysis was performed from November 1 to 24, 2019.

## Results

A total of 2478 individuals (1256 [50.7%] male; 1073 Chinese, 768 Indian, and 637 Malay adults) with a mean (SD) age of 67.6 (5.6) years were included in the final analysis. Those included for analysis had a mean (SD) follow-up period of 6.2 (0.9) years. In comparison, excluded individuals were older, more likely to be Malay, and more likely to have VI (based on better eye), lower AMT scores, no formal education, diabetes, hypertension, history of CVD, CKD, and current smoking status at baseline (eTable 1 in the Supplement). Of the included individuals, 726 had VI at baseline (based on better eye). Compared with individuals without VI at baseline, those with VI at baseline were more likely to be older, female, and Malay; more likely to have lower AMT scores, no formal education, and CKD; and less likely to have alcohol intake at baseline (eTable 2 in the Supplement).

Of the 2478 included individuals, 489 (19.7%) had decreases in AMT scores over 6 years and 1989 did not. Compared with those without decreases in AMT scores over 6 years (542 [27.3%] based on better eye and 1118 [56.2%] based on worse eye; *P* < .001), individuals with decreases in AMT scores were more likely to have VI at baseline (184 [37.6%] based on better eye and 324 [66.3%] based on worse eye; *P* < .001).

Table 1 details the association between baseline vision status and change in AMT score. After baseline age; sex; race/ethnicity; presence of diabetes, hyperlipidemia, hypertension, and CKD; history of CVD; current smoking status; alcohol intake; BMI; educational status; and AMT scores were adjusted for, poorer VA level at baseline was associated with decreases in AMT scores over 6 years (per 0.1 logMAR VA less; β = −0.07; 95% CI, −0.09 to −0.05; *P* < .001 based on the better eye; β = −0.03; 95% CI, −0.04 to −0.02; *P* < .001 based on the worse eye). After adjusting for the same set of covariates, VI at baseline was associated with decreases in AMT scores (β = −0.27; 95% CI, −0.37 to −0.17; *P* < .001 based on the better eye; β = −0.16; 95% CI, −0.26 to −0.07; *P* = .001 based on the worse eye). When further evaluating severity of VI based on the better eye, low vision (β = −0.25; 95% CI, −0.35 to −0.14; *P* < .001) and blindness (β = −1.07; 95% CI, −1.50 to −0.64; *P* < .001) at baseline were significantly associated with decreases in AMT scores compared with normal vision. This finding was similarly observed when evaluating severity of baseline VI based on the worse eye, albeit with slightly smaller effect estimates. In further sensitivity analyses that excluded AMT items 8 and 10 from the aggregate score of AMT, we still observed largely similar associations as the original analyses (eTable 3 and eTable 4 in the Supplement).

**Table 1.** Association Between Baseline Vision Status and Change in Abbreviated Mental Test Score

Baseline vision status^a^	Model 1^b^		Model 2^c^	
	β (95% CI)^**d**^	*P* value	β (95% CI)^**d**^	*P* value
**Based on better eye**^**e**^				
Presenting VA level (per 0.1 logMAR unit)	0.00 (−0.02 to 0.02)	.79	−0.07 (−0.09 to −0.05)	<.001
Visual impairment				
None	1 [Reference]	NA	1 [Reference]	NA
Any	0.01 (−0.10 to 0.12)	.87	−0.27 (−0.37 to −0.17)	<.001
Vision				
Normal	1 [Reference]	NA	1 [Reference]	NA
Low	0.02 (−0.10 to 0.13)	.79	−0.25 (−0.35 to −0.14)	<.001
Blind	−0.15 (−0.61 to 0.31)	.52	−1.07 (−1.50 to −0.64)	<.001
**Based on worse eye**^**f**^				
Presenting VA level (per 0.1 logMAR unit)	0.00 (−0.01 to 0.01)	.52	−0.03 (−0.04 to −0.02)	<.001
Visual impairment				
None	1 [Reference]	NA	1 [Reference]	NA
Any	0.00 (−0.10 to 0.10)	>.99	−0.16 (−0.26 to −0.07)	.001
Vision				
Normal	1 [Reference]	NA	1 [Reference]	NA
Low	0.01 (−0.10 to 0.12)	.84	−0.13 (−0.23 to −0.03)	.008
Blind	−0.07 (−0.27 to 0.12)	.45	−0.41 (−0.59 to −0.23)	<.001

Abbreviations: NA, not applicable; VA, visual acuity.

^a^ Based on US definition: any visual impairment was defined as presenting VA worse than 20/40, low vision was defined as presenting VA worse than 20/40 but better than 20/200, and blindness was defined as presenting VA of 20/200 or worse.

^b^ Adjusted for baseline age, sex, and race/ethnicity.

^c^ Adjusted for baseline age; sex; race/ethnicity; presence of diabetes, hyperlipidemia, hypertension, cardiovascular disease, and chronic kidney disease; current smoking status; alcohol intake; body mass index; educational status; and Abbreviated Mental Test score.

^d^ β Denotes the change in Abbreviated Mental Test score per unit change in exposure variables.

^e^ Includes cases of bilateral low vision, bilateral blindness, and blindness in one eye with low vision in fellow eye.

^f^ Includes cases of unilateral low vision, bilateral low vision, unilateral blindness, bilateral blindness, and blindness in one eye with low vision in fellow eye.

Table 2 details the association between change in vision status (over 6 years) and change in AMT score. After baseline age, sex; race/ethnicity; presence of diabetes, hyperlipidemia, hypertension, and CKD; history of CVD; current smoking status; alcohol intake; BMI; educational status; and AMT score were adjusted for, unchanged or deteriorated VI over time (based on the better eye) were associated with decreases in AMT scores (β = −0.29; 95% CI, −0.40 to −0.18; *P* < .001). Evaluation based on the worse eye demonstrated a similar association (β = −0.20; 95% CI, −0.29 to −0.10; *P* < .001). In further sensitivity analyses that excluded AMT items 8 and 10 from the aggregate score of AMT, we still observed largely similar associations as in the original analyses (eTable 5 and eTable 6 in the Supplement).

**Table 2.** Association Between Change in Vision Status Over 6 Years and Change in Abbreviated Mental Test Score

Change in vision status over 6 y	Model 1^a^		Model 2^b^	
	β (95% CI)^c^	*P* value	β (95% CI)^c^	*P* value
**Based on better eye**				
Remained or improved to normal vision	1 [Reference]	NA	1 [Reference]	NA
Remained or deteriorated to VI^d^	−0.12 (−0.24 to 0.00)	.051	−0.29 (−0.40 to −0.18)	<.001
**Based on worse eye**				
Remained or improved to normal vision	1 [Reference]	NA	1 [Reference]	NA
Remained or deteriorated to VI^d^	−0.05 (−0.15 to 0.06)	.38	−0.20 (−0.29 to −0.10)	<.001

Abbreviations: NA, not applicable; VI, visual impairment.

^a^ Adjusted for baseline age, sex, and ethnicity.

^b^ Adjusted for baseline age; sex; race/ethnicity; presence of diabetes, hyperlipidemia, hypertension, cardiovascular disease, and chronic kidney disease; current smoking status; alcohol intake; body mass index; educational status; and Abbreviated Mental Test score.

^c^ β Denotes the change in Abbreviated Mental Test score per unit change in exposure variables.

^d^ Based on US definition: VI was defined as presenting visual acuity worse than 20/40.

Table 3 details the main causes of VI among individuals with baseline VI and a substantial decrease in AMT score of at least 3 units over 6 years. On the basis of the better eye, the leading causes of VI were undercorrected refractive error (14 [45.2%]) and cataract (11 [35.5%]). Similarly, on the basis of the worse eye, undercorrected refractive error (26 [51%]) and cataract (15 [29.4%]) were the leading causes.

**Table 3.** Main Causes of VI Among Individuals With Baseline VI and Substantial Decrease in AMT Score Over 6 Years

Causes of VI	Participants with baseline VI and decrease in AMT score ≥3 units, No. (%)	
	Based on better eye (n = 31)	Based on worse eye (n = 51)
Undercorrected refractive error	14 (45.2)	26 (51.0)
Cataract	11 (35.5)	15 (29.4)
Diabetic retinopathy	2 (6.5)	2 (3.9)
Posterior capsular opacification	1 (3.2)	1 (2.0)
Pterygium	1 (3.2)	1 (2.0)
Optic atrophy	1 (3.2)	0
Age-related macular degeneration	0	2 (3.9)
Corneal scar	0	1 (2.0)
Macular hole	0	1 (2.0)
Retinal vein occlusion	0	1 (2.0)
Myopic maculopathy	0	1 (2.0)
Others	1 (3.2)	0

Abbreviations: AMT, Abbreviated Mental Test; VI, visual impairment.

Of the 10 items in the AMT (eFigure in the Supplement), item 8 is a vision-related task and required participants to identify the occupation of a person depicted in a picture. Thus, the participant’s response to this item may be particularly sensitive to VI and potentially inflate the originally observed association between VI and cognitive decline. Furthermore, we also evaluated the partial correlation of each AMT item with presenting VA level (eTable 7 in the Supplement) and observed that the association was slightly higher in item 8 compared with most of the other AMT items, although the effect sizes of correlation for all items were generally small. Nevertheless, to rule out this potential bias, we further performed sensitivity analyses that excluded item 8 from the aggregate AMT score (eTable 3 and eTable 5 in the Supplement). The sensitivity analyses demonstrated similar associations as in the original analyses.

It is also possible that participants’ educational level may affect the responses of certain items in the AMT. Thus, we further evaluated the partial correlation of each AMT item with educational level (eTable 8 in the Supplement) and observed that the association was noticeably higher in item 10, which involved memorizing a short phrase. Although educational level was adjusted in our multivariable models, there might still be a residual confounding effect. Furthermore, item 10 had the highest incorrect response rate compared with other items among individuals with VI (eTable 9 in the Supplement). Thus, to rule out that the originally observed finding was not associated with item 10 per se, we also performed another series of sensitivity analyses excluding item 10 from the aggregate AMT score (eTable 4 and eTable 6 in the Supplement). All trends and associations remained consistently similar in these sensitivity analyses.

Because hearing loss and dual sensory loss (hearing impairment and VI) had been reported to be associated with decline in cognitive function,^6,36,37^ we also evaluated the association of self-reported hearing loss at baseline with decline in cognitive function in our study. Nevertheless, we did not find a significant association. We were not able to further perform interaction analysis between baseline hearing loss and VI with decline in cognitive function because of suboptimal model fitting as a result of a limited number of participants with hearing loss. In addition, apart from VI, among the adjusted covariates, age (per year; β = −0.04; 95% CI, −0.04 to −0.03); female sex (β = −0.18; 95% CI, −0.29 to −0.08); Chinese race/ethnicity (β = 0.39; 95% CI, 0.26-0.51); primary education (β = 0.29; 95% CI, 0.17-0.41); O levels or equivalent education (β = 0.35; 95% CI, 0.21-0.50); A levels, polytechnic, diploma, or technical school equivalent (β = 0.27; 95% CI, 0.06-0.47); university education (β = 0.30; 95% CI, 0.07-0.52); BMI (β = −0.01; 95% CI −0.02 to 0.00); and baseline AMT score (β = −0.48; 95% CI, −0.51 to −0.45) were associated with change in AMT score (eTable 10 in the Supplement).

## Discussion

In this study, we observed a 6-year longitudinal association between presenting VI and poorer VA at baseline and decline in cognitive function. Evaluation of presenting VI reflects the habitual vision status of the elderly population. This association was particularly strong for individuals who were blind compared with individuals who had low vision (based on better eye; β = −1.07 vs −0.25). Furthermore, individuals who remained or became visually impaired over 6 years also experienced a greater magnitude of decline in cognitive function, further highlighting the importance of preserving good vision among elderly people. Among individuals with baseline VI and substantial cognitive decline, the leading causes of VI were undercorrected refractive error and cataract, 2 conditions that are easily treatable. To our knowledge, this was the first longitudinal, population-based study of a multiethnic Asian population that prospectively evaluated this association. Our findings may help to better identify elderly people who may be at higher risk for cognitive decline and reemphasize the importance of early detection and management of VI.

We compared our findings with those from other studies,^5,6,7,18,38^ which were mostly conducted in Western populations. Similar to our findings, the Salisbury Eye Evaluation study^5^ in the US reported that poorer presenting VA at baseline was independently associated with lower Mini-Mental State Examination scores during follow-up periods of 2 to 8 years. Likewise, several studies^6,7^ observed increased odds of incident cognitive impairment among individuals with presenting VI at baseline. Specifically, Anstey et al^38^ observed that VA decline in both eyes was associated with memory deterioration. The direction of our findings is similar to these studies,^5,6,7,18,38^ thereby further supporting the association between VI and decline in cognitive function. Nevertheless, the Blue Mountains Eye Study in Australia reported that best-corrected VI (based on better or worse eye) had no association with cognitive deterioration during follow-up of 5 and 10 years.^18^

Several mechanisms have been postulated to potentially explain the association between VI and decline in cognitive function. First, these 2 factors are strongly associated with aging.^39,40,41^ Second, VI may be associated with the reduced frequency of daily activities and ability to perform brain-stimulating activities, such as reading and seeing faces,^8^ thereby reducing cognitive ability because of a lack of sensory stimulus.^9,10,11,12,13^ In this regard, previous studies^42,43^ also found that vision-related interventions, such as the Useful Field of View training, may improve cognitive function among elderly individuals, further indicating the association between vision and cognitive function. Nonetheless, it remains unclear whether the observed association is a direct causal effect or one that is mediated by intermediate factors, such as reduced involvement in social and cognitively stimulating activities owing to VI. Thus, further studies are still required to investigate the potential mediation pathways in this association.

In our study, among individuals with VI at baseline and substantial cognitive decline over 6 years, we identified the leading causes of VI to be undercorrected refractive error and cataract, which are predominantly preventable or treatable. We also observed that individuals with baseline VI that improved to normal vision and those whose vision remained normal over 6 years had significantly lower magnitudes of decline in cognitive function. In this regard, previous studies also reported that cataract surgery with improved visual outcome was associated with improved cognitive performance^44,45^ and with increased gray matter volume in the cortex.^45,46^ Likewise, the Beijing Eye Study observed that individuals with appropriate refractive correction had higher cognitive scores than those without refractive correction.^47^ These findings collectively suggest that the risk of cognitive decline may be ameliorated with appropriate and timely intervention of VI.

### Strengths and Limitations

The strengths of this study include its large size and composition of the 3 major racial/ethnic groups in Asia. In addition, the long follow-up (6 years) of our study provides a unique opportunity to further elucidate the development of cognitive decline, which is a slow and progressive chronic condition. The comprehensive study design also allowed us to provide insights on the main causes of VI among individuals with cognitive decline. This information will be useful in formulating potential interventional strategies.

This study also has limitations. First, although the observed associations were statistically significant, the effect estimates were generally of small magnitude. Thus, the clinical application of our findings needs to be further validated in future studies. Second, the AMT is clinically classified as a screening test,^48^ and its results primarily serve as a proxy for clinically diagnosed cognitive decline. Third, in our study, excluded individuals in general had different profiles compared with included individuals (eTable 1 in the Supplement); thus, potential follow-up bias cannot be entirely ruled out in our sample. Fourth, despite adjusting for a range of potential confounders in our analysis, there might still be a residual confounding effect, particularly because of age and other factors that are associated with aging.

## Conclusions

This prospective, population-based cohort study of a multiethnic Asian population demonstrated that VI was associated with decline in cognitive function over 6 years. Most of these VI cases were attributable to undercorrected refractive error and cataract, which are highly preventable or treatable, therefore further emphasizing the importance of prompt interventions for VI. Holistic measures appear to be needed to manage preventable VI and to reduce the collective burden of both visual and cognitive decline.

## Notes

Supplement. **eFigure.** Locally Validated Abbreviated Mental Test Used in the SEED Study **eTable 1.** Comparison of Baseline Characteristics Between Included and Excluded Subjects **eTable 2.** Comparison of Baseline Characteristics Between Subjects With and Without Visual Impairment at Baseline **eTable 3.** Sensitivity Analysis: Association Between Baseline Vision Status and Change in Abbreviated Mental Test Scores (Excluding Item 8 From Aggregate Score) **eTable 4.** Sensitivity Analysis: Association Between Baseline Vision Status and Change in Abbreviated Mental Test Scores (Excluding Item 10 From Aggregate Score) **eTable 5.** Sensitivity Analysis: Association Between Change in Vision Status (Over 6 Years) and Change in Abbreviated Mental Test Scores (Excluding Item 8 From Aggregate Score) **eTable 6.** Sensitivity Analysis: Association Between Change in Vision Status (Over 6 Years) and Change in Abbreviated Mental Test Scores (Excluding Item 10 From Aggregate Score) **eTable 7.** Partial Correlations Between Each Abbreviated Mental Test Item and Presenting Visual Acuity Level (at Baseline and 6-Year Follow-up Visit) **eTable 8.** Partial Correlations Between Each Abbreviated Mental Test Item and Educational Level (at Baseline and 6-Year Follow-up Visit) **eTable 9.** Number and Proportion of Incorrect Responses for Each Abbreviated Mental Test Item Among Subjects With Visual Impairment (at Baseline and 6-Year Follow-up) **eTable 10.** Associations Between Baseline Factors With Change in Abbreviated Mental Test Score

## References

[1] Bourne RRA, Flaxman SR, Braithwaite T, ; Vision Loss Expert Group Magnitude, temporal trends, and projections of the global prevalence of blindness and distance and near vision impairment: a systematic review and meta-analysis. Lancet Glob Health. 2017;5(9):-. doi:10.1016/S2214-109X(17)30293-0 28779882

[2] United Nations Department of Economic and Social Affairs Key findings and advance tables In: World Population Prospects: The 2017 Revision. United Nations; 2017.

[3] Park SJ, Ahn S, Park KH Burden of visual impairment and chronic diseases. JAMA Ophthalmol. 2016;134(7):778-784. doi:10.1001/jamaophthalmol.2016.1158 27196876

[4] Vos T, Allen C, Arora M, ; GBD 2015 Disease and Injury Incidence and Prevalence Collaborators Global, regional, and national incidence, prevalence, and years lived with disability for 310 diseases and injuries, 1990-2015: a systematic analysis for the Global Burden of Disease Study 2015. Lancet. 2016;388(10053):1545-1602. doi:10.1016/S0140-6736(16)31678-6 27733282 PMC5055577

[5] Zheng DD, Swenor BK, Christ SL, West SK, Lam BL, Lee DJ Longitudinal associations between visual impairment and cognitive functioning: the Salisbury Eye Evaluation Study. JAMA Ophthalmol. 2018;136(9):989-995. doi:10.1001/jamaophthalmol.2018.2493 29955805 PMC6142982

[6] Lin MY, Gutierrez PR, Stone KL, ; Study of Osteoporotic Fractures Research Group Vision impairment and combined vision and hearing impairment predict cognitive and functional decline in older women. J Am Geriatr Soc. 2004;52(12):1996-2002. doi:10.1111/j.1532-5415.2004.52554.x 15571533

[7] Swenor BK, Wang J, Varadaraj V, Vision impairment and cognitive outcomes in older adults: the Health ABC Study. J Gerontol A Biol Sci Med Sci. 2019;74(9):1454-1460. doi:10.1093/gerona/gly244 30358809 PMC6696724

[8] Vu HT, Keeffe JE, McCarty CA, Taylor HR Impact of unilateral and bilateral vision loss on quality of life. Br J Ophthalmol. 2005;89(3):360-363. doi:10.1136/bjo.2004.047498 15722319 PMC1772562

[9] Verghese J, Lipton RB, Katz MJ, Leisure activities and the risk of dementia in the elderly. N Engl J Med. 2003;348(25):2508-2516. doi:10.1056/NEJMoa022252 12815136

[10] Wilson RS, Mendes De Leon CF, Barnes LL, Participation in cognitively stimulating activities and risk of incident Alzheimer disease. JAMA. 2002;287(6):742-748. doi:10.1001/jama.287.6.742 11851541

[11] Verghese J, LeValley A, Derby C, Leisure activities and the risk of amnestic mild cognitive impairment in the elderly. Neurology. 2006;66(6):821-827. doi:10.1212/01.wnl.0000202520.68987.48 16467493 PMC1415273

[12] Clemons TE, Rankin MW, McBee WL; Age-Related Eye Disease Study Research Group Cognitive impairment in the Age-Related Eye Disease Study: AREDS report no. 16. Arch Ophthalmol. 2006;124(4):537-543. doi:10.1001/archopht.124.4.537 16606880 PMC1472655

[13] Swaab DF, Dubelaar EJ, Hofman MA, Scherder EJ, van Someren EJ, Verwer RW Brain aging and Alzheimer’s disease; use it or lose it. Prog Brain Res. 2002;138:343-373. doi:10.1016/S0079-6123(02)38086-5 12432778

[14] Mitoku K, Masaki N, Ogata Y, Okamoto K Vision and hearing impairments, cognitive impairment and mortality among long-term care recipients: a population-based cohort study. BMC Geriatr. 2016;16:112. doi:10.1186/s12877-016-0286-2 27233777 PMC4884419

[15] Chen SP, Bhattacharya J, Pershing S Association of vision loss with cognition in older adults. JAMA Ophthalmol. 2017;135(9):963-970. doi:10.1001/jamaophthalmol.2017.2838 28817745 PMC5710542

[16] Ong SY, Cheung CY, Li X, Visual impairment, age-related eye diseases, and cognitive function: the Singapore Malay Eye study. Arch Ophthalmol. 2012;130(7):895-900. doi:10.1001/archophthalmol.2012.152 22410630

[17] Tay T, Wang JJ, Kifley A, Lindley R, Newall P, Mitchell P Sensory and cognitive association in older persons: findings from an older Australian population. Gerontology. 2006;52(6):386-394. doi:10.1159/000095129 16921251

[18] Hong T, Mitchell P, Burlutsky G, Liew G, Wang JJ Visual impairment, hearing loss and cognitive function in an older population: longitudinal findings from the Blue Mountains Eye Study. PLoS One. 2016;11(1):e0147646. doi:10.1371/journal.pone.0147646 26808979 PMC4726694

[19] World Medical Association World Medical Association Declaration of Helsinki: ethical principles for medical research involving human subjects. JAMA. 2013;310(20):2191-2194. doi:10.1001/jama.2013.281053 24141714

[20] von Elm E, Altman DG, Egger M, Pocock SJ, Gøtzsche PC, Vandenbroucke JP; STROBE Initiative The Strengthening the Reporting of Observational Studies in Epidemiology (STROBE) statement: guidelines for reporting observational studies. Lancet. 2007;370(9596):1453-1457. doi:10.1016/S0140-6736(07)61602-X 18064739

[21] Lavanya R, Jeganathan VS, Zheng Y, Methodology of the Singapore Indian Chinese Cohort (SICC) eye study: quantifying ethnic variations in the epidemiology of eye diseases in Asians. Ophthalmic Epidemiol. 2009;16(6):325-336. doi:10.3109/09286580903144738 19995197

[22] Foong AW, Saw SM, Loo JL, Rationale and methodology for a population-based study of eye diseases in Malay people: the Singapore Malay Eye Study (SiMES). Ophthalmic Epidemiol. 2007;14(1):25-35. doi:10.1080/09286580600878844 17365815

[23] Sabanayagam C, Yip W, Gupta P, Singapore Indian Eye Study-2: methodology and impact of migration on systemic and eye outcomes. Clin Exp Ophthalmol. 2017;45(8):779-789. doi:10.1111/ceo.12974 28472538

[24] Rosman M, Zheng Y, Wong W, Singapore Malay Eye Study: rationale and methodology of 6-year follow-up study (SiMES-2). Clin Exp Ophthalmol. 2012;40(6):557-568. doi:10.1111/j.1442-9071.2012.02763.x 22300454

[25] Tielsch JM, Sommer A, Witt K, Katz J, Royall RM Blindness and visual impairment in an American urban population: the Baltimore Eye Survey. Arch Ophthalmol. 1990;108(2):286-290. doi:10.1001/archopht.1990.01070040138048 2271016

[26] Hodkinson HM Evaluation of a mental test score for assessment of mental impairment in the elderly. Age Ageing. 1972;1(4):233-238. doi:10.1093/ageing/1.4.233 4669880

[27] Qureshi KN, Hodkinson HM Evaluation of a ten-question mental test in the institutionalized elderly. Age Ageing. 1974;3(3):152-157. doi:10.1093/ageing/3.3.152 4463714

[28] Sahadevan S, Lim PP, Tan NJ, Chan SP Diagnostic performance of two mental status tests in the older Chinese: influence of education and age on cut-off values. Int J Geriatr Psychiatry. 2000;15(3):234-241. doi:10.1002/(SICI)1099-1166(200003)15:3<234::AID-GPS99>3.0.CO;2-G 10713581

[29] Ong SY, Ikram MK, Haaland BA, Myopia and cognitive dysfunction: the Singapore Malay Eye Study. Invest Ophthalmol Vis Sci. 2013;54(1):799-803. doi:10.1167/iovs.12-10460 23307956

[30] Annweiler C, Milea D, Whitson HE, Vitamin D insufficiency and cognitive impairment in Asians: a multi-ethnic population-based study and meta-analysis. J Intern Med. 2016;280(3):300-311. doi:10.1111/joim.12491 27037788

[31] Cheung CY, Ong YT, Hilal S, Retinal ganglion cell analysis using high-definition optical coherence tomography in patients with mild cognitive impairment and Alzheimer’s disease. J Alzheimers Dis. 2015;45(1):45-56. doi:10.3233/JAD-141659 25428254

[32] Cheung CY, Ong S, Ikram MK, Retinal vascular fractal dimension is associated with cognitive dysfunction. J Stroke Cerebrovasc Dis. 2014;23(1):43-50. doi:10.1016/j.jstrokecerebrovasdis.2012.09.002 23099042

[33] Ong YT, Hilal S, Cheung CY, Retinal vascular fractals and cognitive impairment. Dement Geriatr Cogn Dis Extra. 2014;4(2):305-313. doi:10.1159/000363286 25298774 PMC4176466

[34] Hilal S, Ikram MK, Saini M, Prevalence of cognitive impairment in Chinese: epidemiology of dementia in Singapore study. J Neurol Neurosurg Psychiatry. 2013;84(6):686-692. doi:10.1136/jnnp-2012-304080 23385846

[35] Levey AS, Stevens LA, Schmid CH, ; CKD-EPI (Chronic Kidney Disease Epidemiology Collaboration) A new equation to estimate glomerular filtration rate. Ann Intern Med. 2009;150(9):604-612. doi:10.7326/0003-4819-150-9-200905050-00006 19414839 PMC2763564

[36] Valentijn SA, van Boxtel MP, van Hooren SA, Change in sensory functioning predicts change in cognitive functioning: results from a 6-year follow-up in the Maastricht Aging Study. J Am Geriatr Soc. 2005;53(3):374-380. doi:10.1111/j.1532-5415.2005.53152.x 15743277

[37] Lin FR, Yaffe K, Xia J, ; Health ABC Study Group Hearing loss and cognitive decline in older adults. JAMA Intern Med. 2013;173(4):293-299. doi:10.1001/jamainternmed.2013.1868 23337978 PMC3869227

[38] Anstey KJ, Luszcz MA, Sanchez L Two-year decline in vision but not hearing is associated with memory decline in very old adults in a population-based sample. Gerontology. 2001;47(5):289-293. doi:10.1159/000052814 11490149

[39] Salthouse TA, Hancock HE, Meinz EJ, Hambrick DZ Interrelations of age, visual acuity, and cognitive functioning. J Gerontol B Psychol Sci Soc Sci. 1996;51(6):317-330. doi:10.1093/geronb/51B.6.P317 8931619

[40] Lindenberger U, Baltes PB Sensory functioning and intelligence in old age: a strong connection. Psychol Aging. 1994;9(3):339-355. doi:10.1037/0882-7974.9.3.339 7999320

[41] Fischer ME, Cruickshanks KJ, Schubert CR, Age-related sensory impairments and risk of cognitive impairment. J Am Geriatr Soc. 2016;64(10):1981-1987. doi:10.1111/jgs.14308 27611845 PMC5073029

[42] Edwards JD, Fausto BA, Tetlow AM, Corona RT, Valdés EG Systematic review and meta-analyses of useful field of view cognitive training. Neurosci Biobehav Rev. 2018;84:72-91. doi:10.1016/j.neubiorev.2017.11.004 29175362

[43] Wolinsky FD, Vander Weg MW, Howren MB, Jones MP, Dotson MM A randomized controlled trial of cognitive training using a visual speed of processing intervention in middle aged and older adults. PLoS One. 2013;8(5):e61624. doi:10.1371/journal.pone.0061624 23650501 PMC3641082

[44] Tamura H, Tsukamoto H, Mukai S, Improvement in cognitive impairment after cataract surgery in elderly patients. J Cataract Refract Surg. 2004;30(3):598-602. doi:10.1016/j.jcrs.2003.10.019 15050255

[45] Lin H, Zhang L, Lin D, Visual restoration after cataract surgery promotes functional and structural brain recovery. EBioMedicine. 2018;30:52-61. doi:10.1016/j.ebiom.2018.03.002 29548900 PMC5952227

[46] Lou AR, Madsen KH, Julian HO, Postoperative increase in grey matter volume in visual cortex after unilateral cataract surgery. Acta Ophthalmol. 2013;91(1):58-65. doi:10.1111/j.1755-3768.2011.02304.x 22103594

[47] Xu L, Wang YX, You QS, Belkin M, Jonas JB Undercorrection of refractive error and cognitive function: the Beijing Eye Study 2011. Acta Ophthalmol. 2014;92(4):e332-e334. doi:10.1111/aos.12265 24119041

[48] Cullen B, O’Neill B, Evans JJ, Coen RF, Lawlor BA A review of screening tests for cognitive impairment. J Neurol Neurosurg Psychiatry. 2007;78(8):790-799. doi:10.1136/jnnp.2006.095414 17178826 PMC2117747

