# Error in Author Surname in Affiliations and Author Contributions

**DOI:** 10.1001/jamanetworkopen.2020.9437

**Published:** 2020-05-20

In the Original Investigation, “Association Between Visual Impairment and Decline in Cognitive Function in a Multiethnic Asian Population,”^1^ published on April 23, 2020, in *JAMA Network Open*, in the Affiliations and Author Contributions, the surname for Zhi Da Soh, MPH, is Soh, not Da Soh. This article has been corrected.

## References

[1] Lim ZW, Chee M-L, Soh ZD, Association between visual impairment and decline in cognitive function in a multiethnic Asian population. JAMA Netw Open. 2020;3(4):e203560. doi:10.1001/jamanetworkopen.2020.3560 32324240 PMC7180417

