# Supplementary material for: Association Between Visual Impairment and Decline in Cognitive Function in a Multiethnic Asian Population
Source: JAMA Netw Open. 2020 Apr 23;3(4):e203560. doi: 10.1001/jamanetworkopen.2020.3560 (PMC7180417; doi:10.1001/jamanetworkopen.2020.3560)
Supplement: Supplement. — eFigure. Locally Validated Abbreviated Mental Test Used in the SEED Study eTable 1. Comparison of Baseline Characteristics Between Included and Excluded Subjects eTable 2. Comparison of Baseline Characteristics Between Subjects With and Without Visual Impairment at Baseline eTable 3. Sensitivity Analysis: Association Between Baseline Vision Status and Change in Abbreviated Mental Test Scores (Excluding Item 8 From Aggregate Score) eTable 4. Sensitivity Analysis: Association Between Baseline Vision Status and Change in Abbreviated Mental Test Scores (Excluding Item 10 From Aggregate Score) eTable 5. Sensitivity Analysis: Association Between Change in Vision Status (Over 6 Years) and Change in Abbreviated Mental Test Scores (Excluding Item 8 From Aggregate Score) eTable 6. Sensitivity Analysis: Association Between Change in Vision Status (Over 6 Years) and Change in Abbreviated Mental Test Scores (Excluding Item 10 From Aggregate Score) eTable 7. Partial Correlations Between Each Abbreviated Mental Test Item and Presenting Visual Acuity Level (at Baseline and 6-Year Follow-up Visit) eTable 8. Partial Correlations Between Each Abbreviated Mental Test Item and Educational Level (at Baseline and 6-Year Follow-up Visit) eTable 9. Number and Proportion of Incorrect Responses for Each Abbreviated Mental Test Item Among Subjects With Visual Impairment (at Baseline and 6-Year Follow-up) eTable 10. Associations Between Baseline Factors With Change in Abbreviated Mental Test Score [file jamanetwopen-3-e203560-s001.pdf]

Supplementary Online Content

Lim ZW, Chee M-L, Da Soh Z, et al. Association between visual impairment and decline in cognitive function in a multiethnic Asian population. *JAMA Netw Open*. 2020;3(4):e203560. doi:10.1001/jamanetworkopen.2020.3560

eFigure. Locally Validated Abbreviated Mental Test Used in the SEED Study

eTable 1. Comparison of Baseline Characteristics Between Included and Excluded Subjects

eTable 2. Comparison of Baseline Characteristics Between Subjects With and Without Visual Impairment at Baseline

eTable 3. Sensitivity Analysis: Association Between Baseline Vision Status and Change in Abbreviated Mental Test Scores (Excluding Item 8 From Aggregate Score)

eTable 4. Sensitivity Analysis: Association Between Baseline Vision Status and Change in Abbreviated Mental Test Scores (Excluding Item 10 From Aggregate Score)

eTable 5. Sensitivity Analysis: Association Between Change in Vision Status (Over 6 Years) and Change in Abbreviated Mental Test Scores (Excluding Item 8 From Aggregate Score)

eTable 6. Sensitivity Analysis: Association Between Change in Vision Status (Over 6 Years) and Change in Abbreviated Mental Test Scores (Excluding Item 10 From Aggregate Score)

eTable 7. Partial Correlations between Each Abbreviated Mental Test Item and Presenting Visual Acuity Level (at Baseline and 6-Year Follow-up Visit)

eTable 8. Partial Correlations Between Each Abbreviated Mental Test Item and Educational Level (at Baseline and 6-Year Follow-up Visit)

eTable 9. Number and Proportion of Incorrect Responses for Each Abbreviated Mental Test Item Among Subjects With Visual Impairment (at Baseline and 6-Year Follow-up)

eTable 10. Associations Between Baseline Factors With Change in Abbreviated Mental Test Score

This supplementary material has been provided by the authors to give readers additional information about their work.

© 2020 Lim ZW et al. *JAMA Network Open*

eFigure. Locally Validated Abbreviated Mental Test Used in the SEED Study

AMT EXAMINATION (For participants aged 60 years and above only)

Score 1 for correct answer.

Please remember the following phrase "37 Bukit Timah Road". I will be asking you to repeat the phrase to me later.

1. *What is the present year? (Western calendar)*

   Right *Wrong*
   ☐1 ☐0

2. *What time is it now? (within 1 hour)*

   Right *Wrong*
   ☐1 ☐0

3. *What is your age?*

   Right *Wrong*
   ☐1 ☐0

4. *What is your date of birth? (Western year +/- mth and day)*

   Right *Wrong*
   ☐1 ☐0

5. *Where are we now?*

   Right *Wrong*
   ☐1 ☐0

6. *What is your home address?*

   Right *Wrong*
   ☐1 ☐0

7. *Who is Singapore's present Prime Minister?*

   Right *Wrong*
   ☐1 ☐0

8. *Show picture of nurse or doctor and ask "What is his/her job?" (picture as below)*

   Right *Wrong*

© 2020 Lim ZW et al. *JAMA Network Open*

☐1 ☐0

9. *Count backwards from 20 to 1*

Right *Wrong*
☐1 ☐0

10. *Please recall the memory phrase mentioned to you in the beginning.*

Right *Wrong*
☐1 ☐0

TOTAL ☐☐

© 2020 Lim ZW et al. *JAMA Network Open*

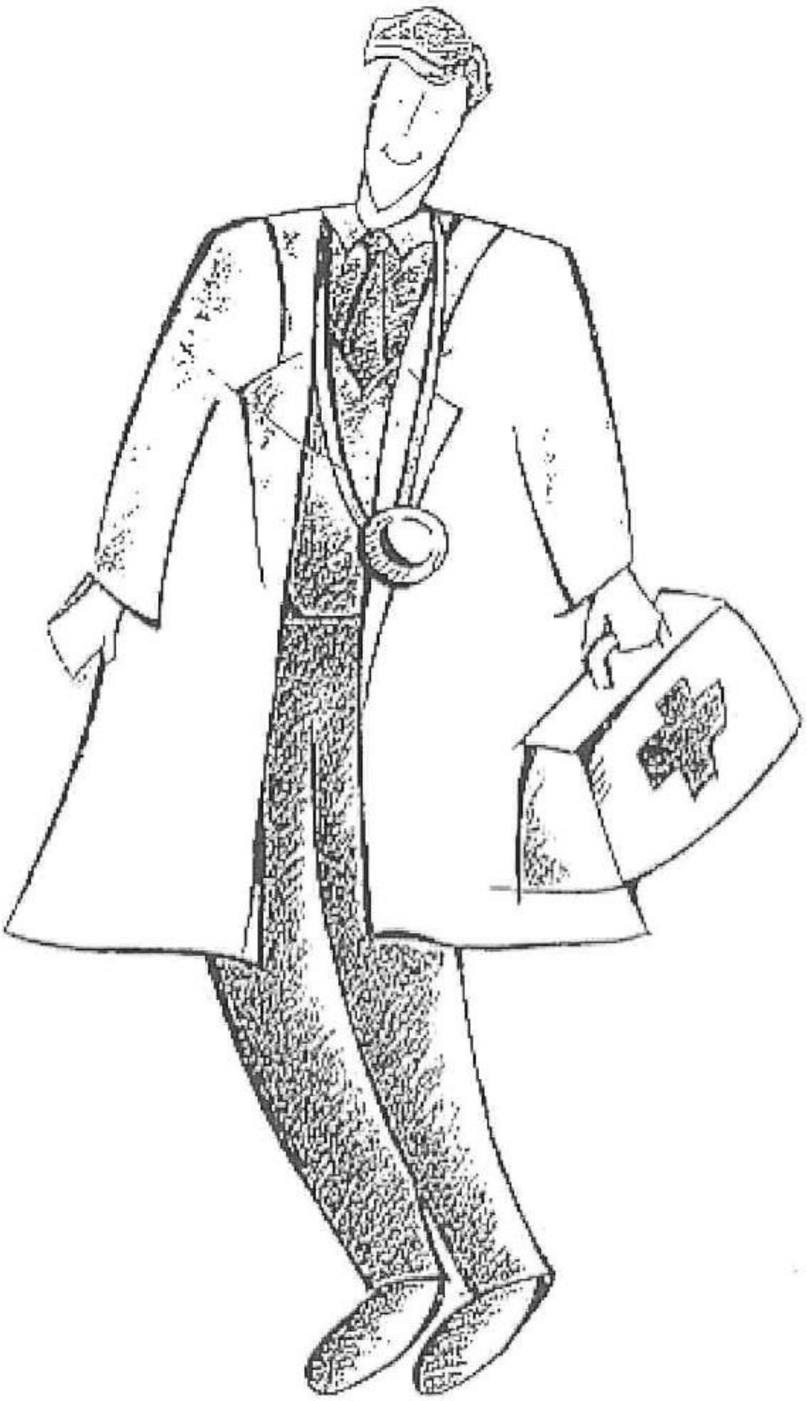

© 2020 Lim ZW et al. *JAMA Network Open*

eTable 1. Comparison of Baseline Characteristics Between Included and Excluded Subjects

Covariate	Excluded (n=1,929)	Included (n=2,478)	P-value[a]
Age, years	70.5 (6.2)	67.6 (5.6)	<0.001
Sex (male), n (%)	1,035 (53.7)	1,256 (50.7)	0.050
Race/ Ethnicity, n (%)			
• Chinese	459 (23.8)	1,073 (43.3)	<0.001
• Indian	598 (31.0)	768 (31.0)	
• Malay	872 (45.2)	637 (25.7)	
Baseline Presenting Visual Impairment (Based on Better Eye), n (%)	838 (43.6)	726 (29.3)	<0.001
Baseline Abbreviated Mental Test Score[b]	8.1 (2.2)	8.8 (1.6)	<0.001
Educational Status, n (%)			
• No Formal Education	887 (46.2)	925 (37.4)	<0.001
• Primary Education	712 (37.1)	853 (34.5)	
• O' Levels or Equivalent	208 (10.8)	410 (16.6)	
• A' Levels/ Polytechnic/ Diploma or Technical School Equivalent	66 (3.4)	157 (6.4)	
• University Education	45 (2.3)	127 (5.1)	
Diabetes Mellitus, n (%)	828 (42.9)	861 (34.7)	<0.001
Hyperlipidaemia, n (%)	992 (54.2)	1,399 (58.3)	0.008
Hypertension, n (%)	1,683 (87.4)	2,001 (80.8)	<0.001
History of Cardiovascular Disease, n (%)	385 (20.1)	342 (13.8)	<0.001
Chronic Kidney Disease, n (%)	594 (32.8)	427 (18.1)	<0.001
Alcohol Intake, n (%)	108 (5.6)	209 (8.4)	<0.001
Current Smoking Status, n (%)	284 (14.8)	270 (10.9)	<0.001
BMI, kg/m^2	25.0 (4.9)	25.1 (4.3)	0.62

Abbreviation: BMI, body mass index

Data presented as mean (standard deviation) or number (percentage), where appropriate.

[a]P-value was estimated based on chi-squared or independent t-test, where appropriate.

[b]Only evaluated among subjects with baseline AMT data.

© 2020 Lim ZW et al. *JAMA Network Open*

eTable 2. Comparison of Baseline Characteristics Between Subjects With and Without Visual Impairment at Baseline

Baseline characteristics	Subjects without baseline VI[a] (n=1,752)	Subjects with baseline VI[a] (n=726)	P-value[b]
Age, years	66.9 (5.3)	69.5 (5.8)	<0.001
Sex (male), n (%)	947 (54.1)	309 (42.6)	<0.001
Race/ Ethnicity, n (%)			
• Chinese	764 (43.6)	309 (42.6)	0.012
• Indian	565 (32.2)	203 (28.0)	
• Malay	423 (24.1)	214 (29.5)	
Baseline Abbreviated Mental Test Score	9.1 (1.3)	8.1 (2.1)	<0.001
Educational Status, n (%)			
• No Formal Education	529 (30.3)	396 (54.7)	<0.001
• Primary Education	627 (35.9)	226 (31.2)	
• O' Levels or Equivalent	340 (19.5)	70 (9.7)	
• A' Levels/ Polytechnic/ Diploma or Technical School Equivalent	139 (8.0)	18 (2.5)	
• University Education	113 (6.5)	14 (1.9)	
Diabetes Mellitus, n (%)	600 (34.2)	261 (36.0)	0.418
Hyperlipidaemia, n (%)	979 (57.4)	420 (60.6)	0.143
Hypertension, n (%)	1,399 (79.9)	602 (82.9)	0.078
History of Cardiovascular Disease, n (%)	233 (13.3)	109 (15.0)	0.255
Chronic Kidney Disease, n (%)	273 (16.2)	154 (22.7)	<0.001
Alcohol Intake, n (%)	161 (9.2)	48 (6.6)	0.035
Current Smoking Status, n (%)	202 (11.5)	68 (9.4)	0.115
BMI, kg/m^2	25.1 (4.1)	25.0 (4.8)	0.77

Abbreviations: VI, visual impairment; BMI, body mass index

Data presented as mean (standard deviation) or number (percentage), where appropriate.

© 2020 Lim ZW et al. *JAMA Network Open*

[a]Defined as presenting visual acuity less than 20/40 in the better eye.

[b]P-value was estimated based on chi-squared or independent t-test, where appropriate.

© 2020 Lim ZW et al. *JAMA Network Open*

eTable 3. Sensitivity Analysis: Association Between Baseline Vision Status and Change in Abbreviated Mental Test Scores (Excluding Item 8 From Aggregate Score)

**Baseline vision status[a]**	**β[b] (95% CI)**	**P-value**
**<u>Based on better eye[c]</u>**		
Presenting VA level (per 0.1 LogMAR unit worse)	-0.06 (-0.08 to -0.04)	<0.001
Visual impairment		
None	1 [Reference]	NA
Any	-0.25 (-0.35 to -0.15)	<0.001
Vision		
Normal	1 [Reference]	NA
Low	-0.22 (-0.32 to -0.13)	<0.001
Blind	-0.97 (-1.37 to -0.56)	<0.001
**<u>Based on worse eye[d]</u>**		
Presenting VA level (per 0.1 LogMAR unit worse)	-0.02 (-0.03 to -0.01)	<0.001
Visual impairment		
None	1 [Reference]	NA
Any	-0.14 (-0.23 to -0.05)	0.002
Vision		
Normal	1 [Reference]	NA
Low	-0.12 (-0.21 to -0.03)	0.010
Blind	-0.31 (-0.48 to -0.14)	<0.001

Abbreviations: NA, not applicable; VA, visual acuity

[a]Based on US definition: any visual impairment was defined as presenting VA worse than 20/40; low vision was defined as presenting VA worse than 20/40 but better than 20/200; and blindness was defined as presenting VA of 20/200 or worse.

[b]Adjusted for baseline age, sex, race/ethnicity, presence of diabetes mellitus, hyperlipidaemia, hypertension, cardiovascular disease, chronic kidney disease, current smoking status, alcohol intake, body mass index, educational status, and Abbreviated Mental Test score.
[b]β denotes the change in Abbreviated Mental Test score per unit change in exposure variables.

[c]Includes cases of bilateral low vision, bilateral blindness, and blindness in one eye with low vision in fellow eye.

[d]Includes cases of unilateral low vision, bilateral low vision, unilateral blindness, bilateral blindness, and blindness in one eye with low vision in fellow eye.

© 2020 Lim ZW et al. *JAMA Network Open*

eTable 4. Sensitivity Analysis: Association Between Baseline Vision Status and Change in Abbreviated Mental Test Scores (Excluding Item 10 From Aggregate Score)

**Baseline vision status[a]**	**β[b] (95% CI)**	**P-value**
**Based on better eye[c]**		
Presenting VA level (per 0.1 LogMAR unit worse)	-0.03 (-0.05 to -0.01)	0.002
Visual impairment		
None	1 [Reference]	NA
Any	-0.13 (-0.22 to -0.05)	0.002
Vision		
Normal	1 [Reference]	NA
Low	-0.13 (-0.22 to -0.05)	0.003
Blind	-0.20 (-0.64 to 0.24)	0.376
**Based on worse eye[d]**		
Presenting VA level (per 0.1 LogMAR unit worse)	-0.01 (-0.02 to 0.00)	0.034
Visual impairment		
None	1 [Reference]	-
Any	-0.11 (-0.19 to -0.03)	0.005
Vision		
Normal	1 [Reference]	-
Low	-0.10 (-0.18 to -0.02)	0.011
Blind	-0.18 (-0.33 to -0.03)	0.023

Abbreviations: NA, not applicable; VA, visual acuity

[a]Based on US definition: any visual impairment was defined as presenting VA worse than 20/40; low vision was defined as presenting VA worse than 20/40 but better than 20/200; and blindness was defined as presenting VA of 20/200 or worse.

[b]Adjusted for baseline age, sex, race/ ethnicity, presence of diabetes mellitus, hyperlipidaemia, hypertension, cardiovascular disease, chronic kidney disease, current smoking status, alcohol intake, body mass index, educational status, and Abbreviated Mental Test score.
[b]β denotes the change in Abbreviated Mental Test score per unit change in exposure variables.

[c]Includes cases of bilateral low vision, bilateral blindness, and blindness in one eye with low vision in fellow eye.

[d]Includes cases of unilateral low vision, bilateral low vision, unilateral blindness, bilateral blindness, and blindness in one eye with low vision in fellow eye.

© 2020 Lim ZW et al. *JAMA Network Open*

eTable 5. Sensitivity Analysis: Association Between Change in Vision Status (Over 6 Years) and Change in Abbreviated Mental Test Scores (Excluding Item 8 From Aggregate Score)

Vision Status	β[a] (95% CI)	P-Value
**Based on better eye**		
Remained or improved to normal vision	1 [Reference]	NA
Remained or deteriorated to VI[b]	-0.24 (-0.34 to -0.13)	<0.001
**Based on worse eye**		
Remained or improved to normal vision	1 [Reference]	NA
Remained or deteriorated to VI[b]	-0.16 (-0.25 to -0.07)	<0.001

Abbreviations: NA, not applicable; VI, visual impairment

[a]Adjusted for baseline age, sex, race/ ethnicity, presence of diabetes mellitus, hyperlipidaemia, hypertension, cardiovascular disease, chronic kidney disease, current smoking status, alcohol intake, body mass index, educational status, and Abbreviated Mental Test score.
[a]β denotes the change in Abbreviated Mental Test score per unit change in exposure variables.

[b]Based on US definition: VI was defined as presenting VA worse than 20/40

eTable 6. Sensitivity Analysis: Association Between Change in Vision Status (Over 6 Years) and Change in Abbreviated Mental Test Scores (Excluding Item 10 From Aggregate Score)

Vision Status	β[a] (95% CI)	P-Value
**<u>Based on better eye</u>**		
Remained or improved to normal vision	1 [Reference]	NA
Remained or deteriorated to VI[b]	-0.13 (-0.23, -0.04)	0.005
**<u>Based on worse eye</u>**		
Remained or improved to normal vision	1 [Reference]	NA
Remained or deteriorated to VI[b]	-0.09 (-0.17, -0.02)	0.017

Abbreviations: NA, not applicable; VI, visual impairment

[a]Adjusted for baseline age, sex, race/ ethnicity, presence of diabetes mellitus, hyperlipidaemia, hypertension, cardiovascular disease, chronic kidney disease, current smoking status, alcohol intake, body mass index, educational status, and Abbreviated Mental Test score.
[a]β denotes the change in Abbreviated Mental Test score per unit change in exposure variables

[b]Based on US definition: VI was defined as presenting VA worse than 20/40

eTable 7. Partial Correlations between Each Abbreviated Mental Test Item and Presenting Visual Acuity Level (at Baseline and 6-Year Follow-up Visit)

	**Baseline visit**		**6-year follow-up**	
**AMT Item**	**Presenting visual acuity (based on worse eye)**	**Presenting visual acuity (based on better eye)**	**Presenting visual acuity (based on worse eye)**	**Presenting visual acuity (based on better eye)**
**1.**	-0.14[b]	-0.18[b]	-0.11[b]	-0.13[b]
**2.**	-0.06[a]	-0.10[b]	-0.07[b]	-0.14[b]
**3.**	-0.07[b]	-0.09[b]	-0.09[b]	-0.10[b]
**4.**	-0.13[b]	-0.18[b]	-0.14[b]	-0.16[b]
**5.**	0.01	-0.04	-0.02	-0.08[b]
**6.**	-0.08[b]	-0.13[b]	-0.09[b]	-0.13[b]
**7.**	-0.11[b]	-0.14[b]	-0.10[b]	-0.10[b]
**8.**	-0.10[b]	-0.17[b]	-0.10[b]	-0.19[b]
**9.**	-0.09[b]	-0.13[b]	-0.09[b]	-0.10[b]
**10.**	-0.06[a]	-0.08[b]	-0.04[a]	-0.07[b]

Abbreviation: AMT, Abbreviated Mental Test

Partial correlation adjusted for age, gender and education.

[a]denotes $P<0.05$

[b]denotes $P<0.001$

© 2020 Lim ZW et al. *JAMA Network Open*

eTable 8. Partial Correlations Between Each Abbreviated Mental Test Item and Educational Level (at Baseline and 6-Year Follow-up Visit)

AMT item	Baseline visit	6-year follow-up
**1.**	0.166[b]	0.158[b]
**2.**	0.063[a]	0.054[a]
**3.**	0.080[b]	0.096[b]
**4.**	0.182[b]	0.148[b]
**5.**	0.068[b]	0.036
**6.**	0.118[b]	0.124[b]
**7.**	0.191[b]	0.115[b]
**8.**	0.126[b]	0.093[b]
**9.**	0.186[b]	0.184[b]
**10.**	0.265[b]	0.252[b]

Abbreviation: AMT, Abbreviated Mental Test

Partial correlation adjusted for age and gender.

[a]denotes P<0.05

[b]denotes P<0.001

© 2020 Lim ZW et al. *JAMA Network Open*

eTable 9. Number and Proportion of Incorrect Responses for Each Abbreviated Mental Test Item Among Subjects With Visual Impairment (at Baseline and 6-Year Follow-up)

	**Baseline visit**		**6-year follow-up**	
**AMT Item**	**Subjects with VI[a] (based on worse eye; N=1,442)**	**Subjects with VI[a] (based on better eye; N=726)**	**Subjects with VI[a] (based on worse eye; N=1,078)**	**Subjects with VI[a] (based on better eye; N=594)**
**1.**	213 (14.8)	151 (20.8)	181 (16.8)	136 (22.9)
**2.**	46 (3.2)	36 (5.0)	43 (4.0)	37 (6.2)
**3.**	97 (6.7)	66 (9.1)	88 (8.2)	65 (10.9)
**4.**	233 (16.2)	165 (22.7)	177 (16.4)	128 (21.5)
**5.**	41 (2.8)	29 (4.0)	42 (3.9)	30 (5.1)
**6.**	125 (8.7)	89 (12.3)	114 (10.6)	77 (13.0)
**7.**	272 (18.9)	183 (25.2)	105 (9.7)	70 (11.8)
**8.**	119 (8.3)	89 (12.3)	102 (9.5)	82 (13.8)
**9.**	255 (17.7)	172 (23.7)	197 (18.3)	134 (22.6)
**10.**	689 (47.8)	386 (53.2)	504 (46.8)	313 (52.7)

Abbreviation: VI, visual impairment

[a]Based on US definition: VI was defined as presenting VA worse than 20/40

© 2020 Lim ZW et al. *JAMA Network Open*

eTable 10. Associations Between Baseline Factors With Change in Abbreviated Mental Test Score

Baseline Characteristics	β (95% CI)[a]	P-value
Age, per year	-0.04 (-0.04, -0.03)	<0.001
Sex (female)	-0.18 (-0.29, -0.08)	0.001
Race/ Ethnicity		
• Malay	1 [Reference]	NA
• Indian	0.05 (-0.07, 0.18)	0.402
• Chinese	0.39 (0.26, 0.51)	<0.001
Educational Status		
• No Formal Education	1 [Reference]	NA
• Primary Education	0.29 (0.17, 0.41)	<0.001
• O' Levels or Equivalent	0.35 (0.21, 0.50)	<0.001
• A' Levels/ Polytechnic/ Diploma or Technical School Equivalent	0.27 (0.06, 0.47)	0.010
• University Education	0.30 (0.07, 0.52)	0.009
Diabetes Mellitus		
• Yes	-0.05 (-0.15, 0.05)	0.312
Hyperlipidaemia		
• Yes	0.01 (-0.08, 0.11)	0.778
Hypertension		
• Yes	0.05 (-0.07, 0.17)	0.409
Cardiovascular Disease		
• Yes	-0.03 (-0.17, 0.11)	0.677
Chronic Kidney Disease		
• Yes	0.00 (-0.12, 0.12)	0.997
Alcohol Intake		
• Yes	0.02 (-0.15, 0.19)	0.857
Current Smoking Status		
• Yes	-0.15 (-0.30, 0.00)	0.054
BMI (kg/m^2)	-0.01 (-0.02, 0.00)	0.04
Baseline Abbreviated Mental Test Score	-0.48 (-0.51, -0.45)	<0.001

Abbreviation: NA, not applicable; BMI, body mass index

[a]Adjusted for baseline age, sex, race/ ethnicity, presence of diabetes mellitus, hyperlipidaemia, hypertension, cardiovascular disease, chronic kidney disease, current smoking status, alcohol intake, body mass index, educational status, Abbreviated Mental Test score and presenting VI (based on better eye).
[a]β denotes the change in Abbreviated Mental Test score per unit change in exposure variables.

© 2020 Lim ZW et al. *JAMA Network Open*
